# Relationships Between Instructor Leadership Styles, Self-Efficacy, and Exercise Adherence Intention Among Recreational Badminton Participants

**DOI:** 10.3390/bs16101788

**Published:** 2026-10-01

**Authors:** Sheng Yen Lee, Han Sung Lee, Sareena Hanim Hamzah, Zulkarnain Jaafar, Se Ik Park

**Affiliations:** 1 Department of Sport Healthcare, Gyeongsang National University, Jinju-si 52725, Republic of Korea; sylee7@gnu.ac.kr; 2 Department of Leisure Sports, Seoil University, 28 Yongmasan-ro 90-gil, Jungnang-gu, Seoul 02192, Republic of Korea; hans3090@naver.com; 3 Faculty of Sports and Exercise Science, Universiti Malaya, Kuala Lumpur 50603, Malaysia; sareena@um.edu.my (S.H.H.); zulkarnainj@um.edu.my (Z.J.)

**Keywords:** sport psychology, self-efficacy, exercise adherence intention, leadership style, recreational badminton, exercise behavior

## Abstract

This study examined the relationships between perceived badminton instructors’ leadership styles (training and instruction, democratic, positive feedback, and social support behaviors), self-efficacy, and exercise adherence intention. Data were collected through a questionnaire survey from late August to early October 2025, yielding 186 valid responses from 210 distributed questionnaires. Frequency and reliability analyses were conducted and structural equation modeling was performed. Training and instruction behavior was positively related to self-efficacy, whereas democratic, positive feedback, and social support behaviors were not significantly related to self-efficacy. Positive feedback behavior was positively related to exercise adherence intention, while training and instruction, democratic, and social support behaviors were not significantly related to exercise adherence intention. Self-efficacy was positively related to exercise adherence intention. Self-efficacy significantly mediated the relationship between training and instruction behavior and exercise adherence intention, whereas no significant mediation was found for democratic, positive feedback, or social support behaviors. This study identifies the structural relationships between perceived badminton instructors’ leadership styles, self-efficacy, and exercise adherence intention. The findings provide practical implications and evidence for developing effective coaching strategies to support exercise adherence intention among recreational badminton participants.

## 1. Introduction

Rapid advances in science and technology, along with economic growth and increased income, have significantly contributed to material prosperity in modern society. As interest in well-being and quality of life has increased (S. Y. Lee, 2018), physical activity has become an important means of maintaining health and promoting daily vitality (M. J. Lee, 2013).

In Korea, the implementation of the five-day and 52 h workweek in 2018 increased leisure time and opportunities for participation in sports and physical activities (Choi, 2020). Among various leisure sports, badminton has emerged as one of the most popular activities across all age groups. Badminton requires simple equipment and can be played in almost any setting, regardless of season or location. It also provides opportunities for stress relief and social interaction (Son & Kim, 2016). According to the 2021 National Survey of Sports Participation, badminton ranked among the top ten most popular sports in Korea and third among sports commonly practiced through sports clubs (Ministry of Culture, Sports and Tourism, 2021). Furthermore, in 2022, a total of 2795 badminton clubs were operating nationwide (Korea Badminton Association, 2022). Through these clubs, participants ranging from beginners to advanced players receive systematic badminton instruction to improve their skills and participate in higher-level competitions (Nam, 2021).

Previous studies have shown that coaches play a critical role in improving elite athletes’ performance. In particular, numerous studies have examined which coaching styles effectively guide athletes and produce successful performance outcomes across sports. However, while elite athletes typically participate to improve performance, recreational participants engage in sport for diverse reasons and vary widely in skill level. Accordingly, elite athletes generally need coaching focused on improving performance and achieving better results, whereas recreational participants may need greater personal support and emphasis on enjoyment, skill development, and continued participation. This fundamental distinction underscores the importance of examining leadership styles in recreational sport contexts. For recreational badminton participants, instructors should provide goals and motivation aligned with participants’ reasons for participation. Identifying specific leadership styles that foster continued interest and encourage active participation is therefore important.

One of the most influential frameworks in sport leadership is Chelladurai and Saleh’s (1980) multidimensional model of leadership. Studies by Horne and Carron (1985), Weiss and Friedrichs (1986), and Schliesman (1987) have also highlighted the importance of coaching behaviors in effective athlete development (S. Y. Lee et al., 2020). According to the multidimensional model of leadership, sport leadership consists of five dimensions: training and instruction, democratic, positive feedback, social support, and autocratic behaviors (Chelladurai & Saleh, 1980). Because recreational badminton participants have different goals and skill levels, this model is suitable for examining different instructor leadership behaviors in relation to their needs and participation.

Previous studies have reported that coaches’ leadership styles significantly influence athlete outcomes, including performance improvement. However, leadership styles and their effects may differ depending on the specific sport, participant characteristics, participation goals, and skill levels. Although athletic performance is the primary objective for elite athletes, recreational participants may place greater value on perceived exercise benefits when deciding whether to continue participating. Therefore, understanding how badminton instructors’ leadership styles are related to self-efficacy may provide important insights into promoting continued sport participation and active involvement among recreational participants.

Self-efficacy refers to an individual’s belief in their ability to successfully perform a task or overcome challenges through personal effort and capability (Bandura, 1977). Gully et al. (2002) reported that self-efficacy is closely associated with successful task performance and plays a critical role in sustaining effort and continued participation. Therefore, examining how badminton participation relates to self-efficacy is important. Understanding participants’ levels of self-efficacy may also provide crucial insights into exercise outcomes and exercise adherence intention. Thus, self-efficacy may mediate the relationship between instructor leadership styles and exercise adherence intention because leadership behaviors may be associated with greater confidence in exercise, which may support participants’ intention to continue exercising.

Previous studies related to this topic can be categorized into three main areas. First, studies on badminton instructors’ leadership styles have primarily focused on relationships among variables such as athletic performance, exercise commitment, and exercise adherence intention (Y. S. Cho & Choi, 2016; Fouraki et al., 2020; Khaskheli et al., 2019; Nam, 2021; Su et al., 2024). However, these studies have paid limited attention to recreational participants’ perceptions of exercise outcomes and participation experiences. Second, studies on self-efficacy among badminton participants have examined how badminton participation affects self-efficacy (G. S. Cho & Lee, 2019; Fatima et al., 2025; Hidayat et al., 2023; Ilham & Dimyati, 2021; Lim & Kang, 2011; Park et al., 2021). Despite the close interaction between instructors and participants, the specific dynamics of instructor–participant relationships have received limited attention in this context. Third, although studies on leisure satisfaction among badminton participants and psychological well-being derived from badminton participation provide evidence regarding participation benefits, they do not directly examine participants’ intentions to continue participating in relation to interactions with instructors (Jung & Jin, 2021; M. L. Kim, 2022, H. J. Kim, 2023). Overall, these studies suggest that instructor leadership styles may be related to participants’ self-efficacy, which may, in turn, be related to their exercise adherence intention.

To date, few studies have specifically examined the relationships among badminton instructors’ leadership styles, self-efficacy, and exercise adherence intention in recreational badminton contexts. However, it remains unclear how different instructor leadership styles are related to exercise adherence intention and whether self-efficacy plays a mediating role in these relationships. Therefore, it is necessary to investigate how badminton instructors’ leadership styles are related to self-efficacy and exercise adherence intention.

Accordingly, this study aims to identify perceived badminton instructors’ leadership styles and examine the mediating role of self-efficacy in the relationship between leadership styles and exercise adherence intention. Through this investigation, the study seeks to provide practical evidence for developing effective coaching strategies and encouraging continued participation in badminton, thereby contributing to participants’ physical, psychological, and social well-being.

## 2. Theoretical Background

### 2.1. Leadership Styles of Badminton Instructors

In the field of sports coaching, a systematic analysis of instructors’ leadership styles is necessary. Chelladurai and Saleh (1980) proposed one of the most influential frameworks for understanding coaching behaviors. According to the multidimensional model of leadership, leadership styles can be classified into five dimensions: training and instruction, democratic, positive feedback, social support, and autocratic behaviors. This model provides a systematic framework for understanding coaching behaviors in sport settings and suggests that different leadership styles may shape athletes’ psychological responses and, ultimately, affect sport performance.

Research on leadership styles has been widely conducted in elite sports, whereas studies in recreational sport settings remain relatively limited. Y. N. Kim and Jung (2016) reported that badminton coaches’ leadership styles had significant effects on athletes’ life satisfaction and exercise adherence intention, highlighting the critical role of coaches in promoting continued sport participation. Conversely, studies involving recreational sport participants, such as those by Nam (2021) and G. S. Cho and Lee (2019), have applied the multidimensional model of leadership to examine athletic performance and exercise adherence intention. Democratic behavior may support self-efficacy by encouraging participants to take an active role in decision-making, while positive feedback behavior may strengthen participants’ confidence by recognizing their effort and performance (Nam, 2021). However, relatively few studies have investigated exercise adherence intention through psychological mechanisms such as self-efficacy among general sport participants.

### 2.2. Self-Efficacy

Self-efficacy refers to an individual’s belief in their ability to successfully perform a specific task or behavior (Bandura, 1977). According to Bandura’s social cognitive theory (SCT), self-efficacy can be shaped by social and environmental factors, which may influence an individual’s motivation and continued behavior. Individuals with higher levels of self-efficacy are more likely to exert greater effort, persist when facing difficulties, and continue engaging in behaviors despite challenges. Accordingly, this study focuses on general self-efficacy, which reflects an individual’s broader belief in their ability to manage difficulties and perform necessary behaviors successfully. Such beliefs can support persistence and sustained effort across situations and thus be related to exercise adherence intention.

Self-efficacy has also been widely examined in exercise and recreational sport participation contexts (S. Y. Lee et al., 2024). Individuals with high self-efficacy are more likely to demonstrate the effort and perseverance necessary for sustained participation in physical activity (H. J. Lee, 2011). Previous studies have identified self-efficacy as an important predictor of motivation and exercise adherence intention in sport participation. In a study of university students participating in leisure sports, H. J. Lee (2011) reported that individuals with higher intrinsic motivation and stronger self-efficacy showed significantly greater intentions to continue participating in exercise. Similarly, Y. S. Cho and Choi (2016), in a study of adolescent judo athletes, found that parental social support increased athletes’ self-efficacy, which, in turn, mediated improvements in exercise adherence intention. These previous studies suggest that instructor leadership behaviors may be related to exercise adherence intention through participants’ self-efficacy.

### 2.3. Exercise Adherence Intention

Exercise adherence intention refers to an individual’s intention to continue participating in exercise or sports activities in the future (Ajzen, 1991). According to the theory of planned behavior (TPB), behavioral intention is the most immediate determinant of actual behavior and is influenced by three factors: attitudes, subjective norms, and perceived behavioral control (Ajzen, 1991). Individuals are more likely to continue exercising when they hold positive attitudes toward exercise, perceive supportive social environments, and feel a sense of control over their behavior. Self-efficacy is conceptually related to perceived behavioral control, as both concern an individual’s perceived ability to perform a behavior (Ajzen, 1991; Bandura, 1977). From this perspective, SCT explains how social and environmental factors, including instructor leadership, may relate to participants’ self-efficacy, while TPB explains how self-efficacy may relate to exercise adherence intention (Ajzen, 1991; Bandura, 1977). Together, SCT and TPB provide a theoretical basis for examining the direct relationship between instructor leadership and exercise adherence intention and the indirect relationship through self-efficacy.

S. Y. Lee (2017) examined the effects of service quality and customer loyalty on exercise adherence intention among sport center users and reported that both the service environment and participants’ psychological perceptions positively influenced exercise adherence intention. Specifically, high-quality facilities and instructor services were found to foster customer attachment, which subsequently contributed to continued participation in exercise. Similarly, S. Y. Lee et al. (2019) found that service quality in sport climbing facilities positively influenced exercise adherence intention through psychological factors such as customer satisfaction and customer trust. These findings suggest that psychological factors play an important role in promoting continued participation among recreational sport participants.

Overall, exercise adherence intention can be understood as a concept shaped not only by participation habits but also by participants’ psychological attitudes, social environments, and service experiences. Therefore, examining the relationships among leadership styles, self-efficacy, and exercise adherence intention has practical relevance for developing effective coaching strategies that encourage continued participation among badminton participants (Ajzen, 1991; S. Y. Lee, 2017; S. Y. Lee et al., 2019).

Based on these findings, this study examines the relationship between badminton instructors’ leadership styles and self-efficacy, identifies which leadership styles are positively associated with self-efficacy, and investigates the relationship between self-efficacy and exercise adherence intention. By examining the mediating role of self-efficacy in the relationship between leadership styles and exercise adherence intention, this study seeks to provide empirical evidence on these relationships among recreational badminton participants.

### 2.4. Hypotheses and Research Model

**Figure 1:**
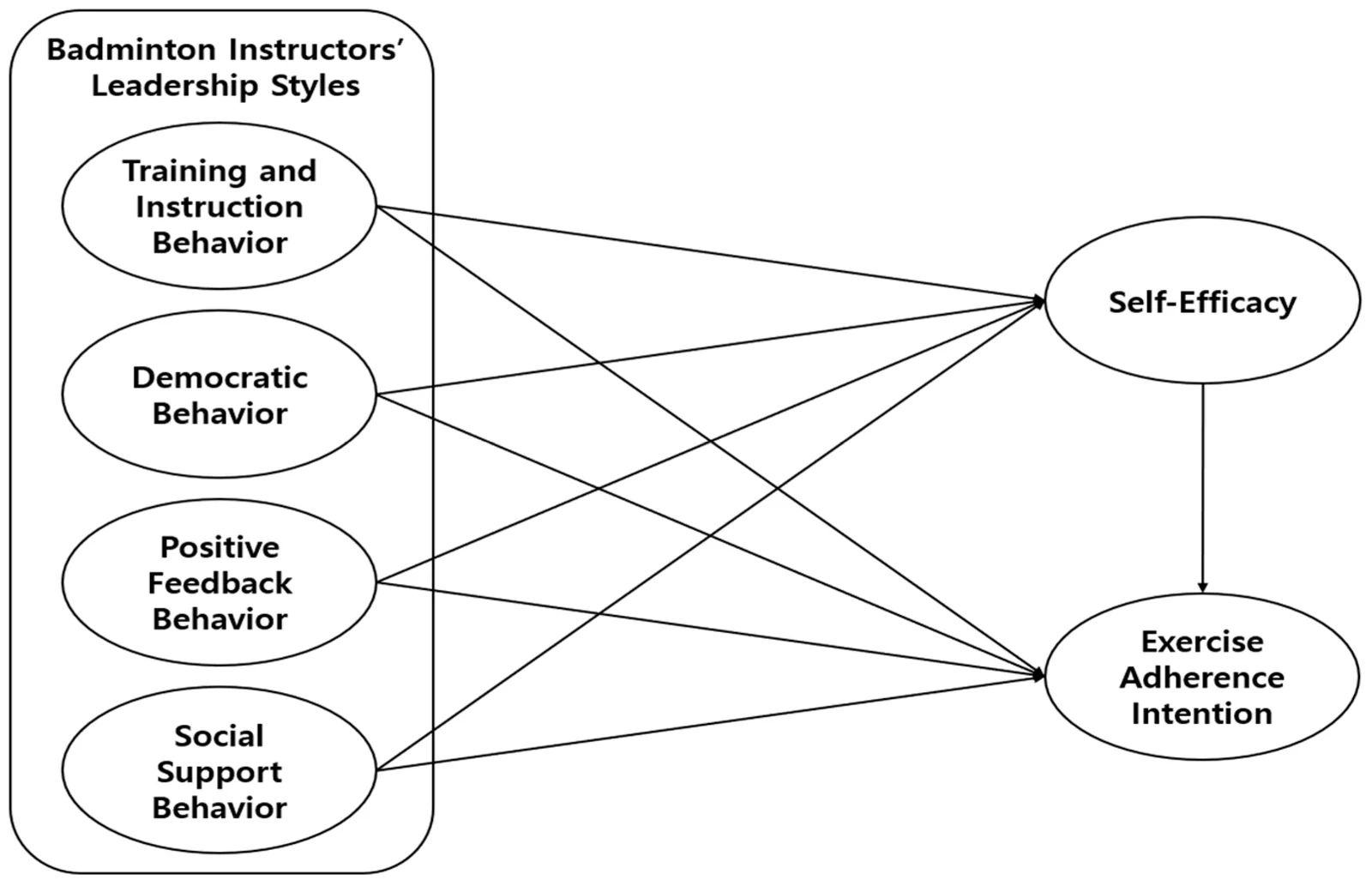
Research model.

To address the research gaps identified above, the following hypotheses were developed, and the research model was established as shown in Figure 1.

Nam (2021) reported a relationship between instructors’ leadership styles and self-efficacy among recreational badminton participants. Based on this finding, the following hypothesis was proposed:

**H1.**  *Badminton instructors’ leadership styles (training and instruction, democratic, positive feedback, and social support behaviors) will have a positive relationship with self-efficacy.*

Y. N. Kim and Jung (2016) reported a relationship between badminton coaches’ leadership styles and exercise adherence intention. Based on this finding, the following hypothesis was proposed:

**H2.**  *Badminton instructors’ leadership styles (training and instruction, democratic, positive feedback, and social support behaviors) will have a positive relationship with exercise adherence intention.*

H. J. Lee (2011) reported a positive relationship between self-efficacy and continued participation intention. Based on this finding, the following hypothesis was proposed:

**H3.**  *Self-efficacy will have a positive relationship with exercise adherence intention.*

Nam (2021) identified the mediating role of self-efficacy in the relationship between leadership styles and performance among recreational badminton participants. Y. S. Cho and Choi (2016) also identified the mediating role of self-efficacy in relation to exercise adherence intention. Based on these findings, the following hypothesis was proposed:

**H4.**  *Self-efficacy will mediate the relationship between badminton instructors’ leadership styles (training and instruction, democratic, positive feedback, and social support behaviors) and exercise adherence intention.*

## 3. Materials and Methods

### 3.1. Participants

The target population comprised adult recreational badminton participants currently taking badminton lessons under an instructor’s guidance. Competitive badminton athletes were excluded from the study. No other inclusion or exclusion criteria were applied. Convenience sampling, a non-probability sampling technique, was employed.

Data were collected through a questionnaire survey conducted from late August to early October 2025. A total of 210 participants taking badminton lessons were invited to participate in the study. The researchers and research assistants visited sports facilities, confirmed participants’ involvement in badminton activities, explained the survey purpose and procedures, and encouraged voluntary participation.

After excluding incomplete or unreliable responses, 186 questionnaires were retained for the final analysis. G*Power 3.1.9.7 was used to determine the required sample size (Faul et al., 2009). With the effect size set at f^2^ = 0.15, alpha at 0.05, and statistical power at 0.95, the minimum required sample size was 138. The final sample of 186 exceeded the required sample size.

Among the final sample, 118 participants (63.4%) were male, and 68 (36.6%) were female. Participants in their 30s represented the largest age group. Regarding participation frequency, the largest proportion of participants (34.4%) reported playing badminton one to two times per week. Additionally, the largest proportion of respondents reported participating for 1 to 2 h per session. Detailed demographic characteristics of the participants are presented in Table 1.

### 3.2. Measures

To examine the relationships between perceived badminton instructors’ leadership styles, self-efficacy, and exercise adherence intention, questionnaires validated in previous studies were adopted and modified for this study. All items were rated on a five-point Likert scale. In the present study, instructor leadership styles refer to participants’ perception of instructor leadership behaviors.

Leadership styles were measured using the Leadership Scale for Sports, originally developed by Chelladurai and Saleh (1980) and later adapted by S. Y. Lee et al. (2020). The instrument was modified for this study. The scale consisted of five sub-dimensions: training and instruction (6 items), democratic (6 items), positive feedback (5 items), social support (6 items), and autocratic behaviors (6 items), totaling 29 items. All five dimensions were initially included in the measurement. However, confirmatory factor analysis (CFA) indicated that the autocratic behavior dimension did not demonstrate satisfactory construct validity. Consequently, the autocratic behavior dimension was excluded, and the final measurement model consisted of 13 items across four dimensions: training and instruction (4 items), democratic (3 items), positive feedback (3 items), and social support behaviors (3 items).

Self-efficacy items were adapted with reference to Schunk (1984), McAuley (1993), S. Y. Lee et al. (2024), and Bandura’s (1977) concept of self-efficacy. Exercise adherence intention was measured using items adapted from S. Y. Lee (2018). The modified items were reviewed by two university professors with expertise in sport psychology for appropriateness and clarity. No formal content validity index was calculated. Following CFA, all six self-efficacy items and three exercise adherence intention items were retained in the final measurement model.

### 3.3. Data Analysis

Data were analyzed using SPSS 29.0 and AMOS 31.0.

Frequency analysis was conducted to examine participants’ demographic characteristics. CFA was performed to evaluate the measurement model and assess construct, convergent, and discriminant validity. Structural equation modeling (SEM) was then conducted to test the hypothesized relationships among the latent variables. Finally, the mediating effect of self-efficacy on the relationship between badminton instructors’ leadership styles and exercise adherence intention was examined using 5000 bootstrap samples with bias-corrected 95% confidence intervals.

## 4. Results

### 4.1. Validity and Reliability Analyses

To examine the relationships between badminton instructors’ leadership styles, self-efficacy, and exercise adherence intention, validity and reliability analyses were conducted for each construct. CFA was performed to assess the construct validity of the proposed variables, and only items that demonstrated acceptable validity and model fit were retained for the final analysis.

Model fit was evaluated using chi-square (χ^2^), normed chi-square (CMIN/df), root mean square residual (RMR), root mean square error of approximation (RMSEA), Tucker–Lewis index (TLI), comparative fit index (CFI), goodness-of-fit index (GFI), and adjusted goodness-of-fit index (AGFI). Based on the criteria suggested by Song (2015), model fit was considered acceptable when RMR was below 0.05, RMSEA was below 0.10, and CFI, normed fit index (NFI), and GFI were above 0.80. Items with factor loadings below 0.50 were removed from the analysis. The full measurement model, including all latent variables, demonstrated an acceptable fit (χ^2^ = 338.787, df = 194, *p* < 0.001, CMIN/df = 1.746, GFI = 0.863, AGFI = 0.821, CFI = 0.950, NFI = 0.892, RMR = 0.038, and RMSEA = 0.064). Detailed CFA results for each construct are presented in Table 2, Table 3 and Table 4. Additionally, reliability analysis showed that all sub-dimensions of the study variables had Cronbach’s alpha values above 0.70.

Multicollinearity was examined among the leadership dimensions, with VIF values ranging from 2.058 to 3.343 and tolerance values ranging from 0.299 to 0.486, indicating no serious multicollinearity concerns.

#### 4.1.1. Leadership Styles of Badminton Instructors

The CFA for badminton instructors’ leadership styles indicated acceptable model fit (RMSEA = 0.077, RMR = 0.034, GFI = 0.904, AGFI = 0.851, CFI = 0.960, and NFI = 0.927). Four sub-dimensions were retained for the final analysis: training and instruction, democratic, positive feedback, and social support behaviors.

Reliability analysis showed Cronbach’s alpha values of 0.859 for training and instruction behavior, 0.844 for democratic behavior, 0.879 for positive feedback behavior, and 0.847 for social support behavior, indicating satisfactory reliability. The results of the factor and reliability analyses for leadership styles are presented in Table 2.

#### 4.1.2. Self-Efficacy

The CFA for self-efficacy indicated an acceptable model fit (RMSEA = 0.070, RMR = 0.017, GFI = 0.971, AGFI = 0.933, CFI = 0.987, and NFI = 0.973). Reliability analysis yielded a Cronbach’s alpha value of 0.903, indicating satisfactory internal consistency. The results of the factor and reliability analyses for self-efficacy are presented in Table 3.

#### 4.1.3. Exercise Adherence Intention

Exercise adherence intention was measured using three items. The CFA resulted in a saturated model (χ^2^ = 0.000, df = 0), indicating that model fit indices were not applicable. All standardized factor loadings exceeded 0.80, ranging from 0.846 to 0.917, and were statistically significant (*p* < 0.001). Reliability analysis yielded a Cronbach’s α of 0.921, indicating excellent internal consistency. The results of the CFA and reliability analysis for exercise adherence intention are presented in Table 4.

### 4.2. Correlation Analysis

Table 5 presents the correlation matrix for the study constructs. The construct reliability (CR) values ranged from 0.845 to 0.922, and the average variance extracted (AVE) values ranged from 0.610 to 0.798. All constructs exceeded the recommended thresholds for CR (>0.70) and AVE (>0.50), indicating satisfactory convergent validity. Discriminant validity was assessed by comparing correlations between constructs with the square root of the AVE (√AVE) for each construct. All correlations were lower than the corresponding √AVE values, satisfying the discriminant validity criterion suggested by Song (2015). Therefore, the measurement model was considered appropriate for subsequent structural model analysis and hypothesis testing.

### 4.3. Hypothesis Testing

Path analysis was conducted to test the hypotheses regarding the relationships among badminton instructors’ leadership styles, self-efficacy, and exercise adherence intention.

For H1, training and instruction behavior had a significant positive relationship with self-efficacy (β = 0.396, *p* = 0.001), whereas democratic behavior, positive feedback behavior, and social support behavior were not significantly related to self-efficacy.

For H2, among the sub-dimensions of badminton instructors’ leadership styles, positive feedback behavior had a significant positive relationship with exercise adherence intention (β = 0.521, *p* = 0.016), whereas training and instruction behavior, democratic behavior, and social support behavior were not significantly related to exercise adherence intention.

H3 was supported; self-efficacy had a significant positive relationship with exercise adherence intention (β = 0.358, *p* < 0.001).

The results for Hypotheses 1–3 are presented in Table 6.

The complete- and partial-mediation models were compared to determine the appropriate structural model. The complete-mediation model yielded χ^2^ = 356.091 (df = 198), whereas the partial-mediation model yielded χ^2^ = 338.787 (df = 194). The chi-square difference test was significant (Δχ^2^ = 17.304, Δdf = 4, *p* = 0.002), indicating better fit for the partial-mediation model. Therefore, the partial-mediation model was retained for hypothesis testing.

**Figure 2:**
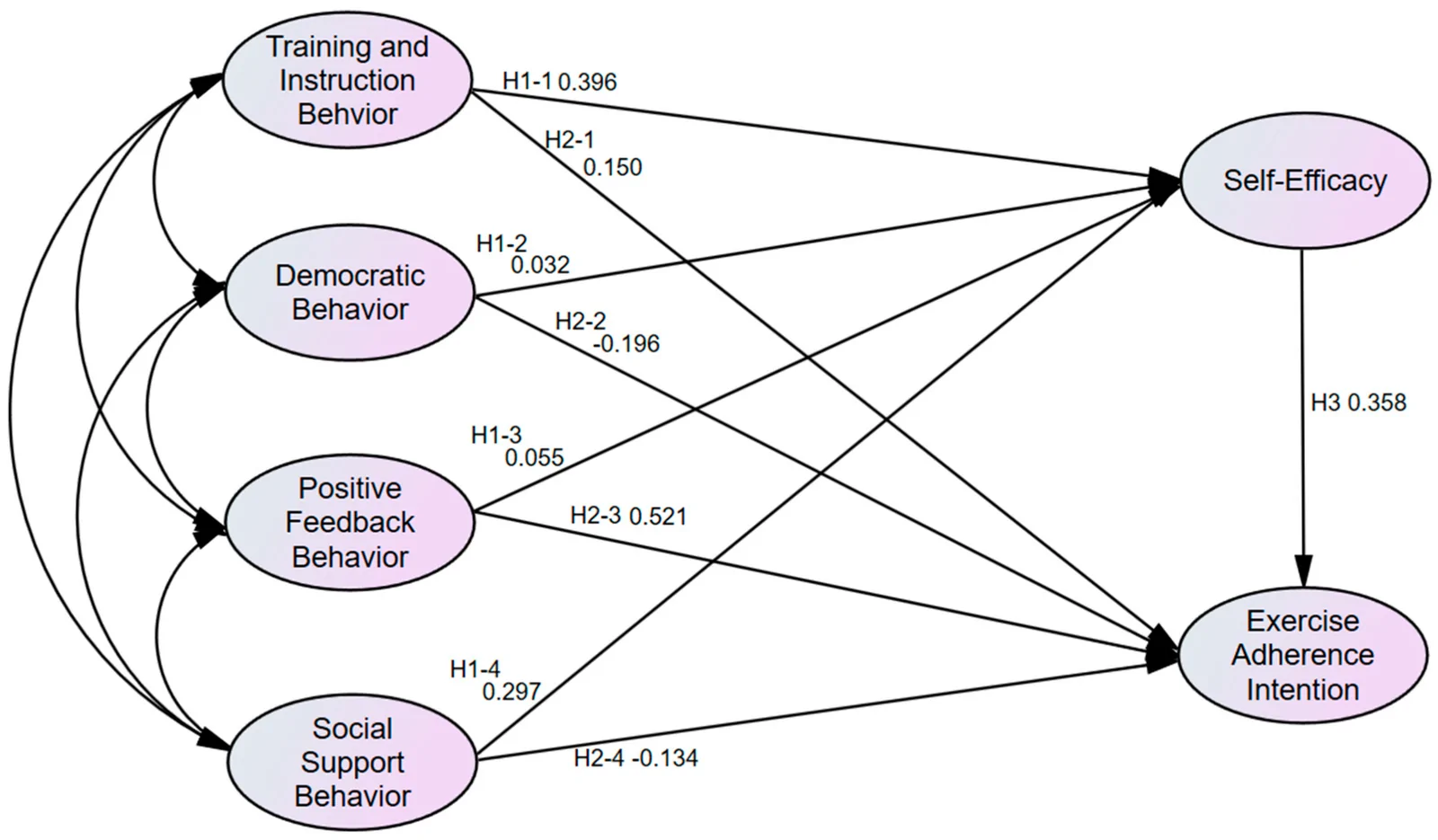
Validated Model for Hypotheses 1–3.

The overall structural model demonstrated an acceptable fit to the data (χ^2^ = 338.787, df = 194, *p* < 0.001, CMIN/df = 1.746, GFI = 0.863, AGFI = 0.821, CFI = 0.950, TLI = 0.941, NFI = 0.892, RMR = 0.038, SRMR = 0.046, and RMSEA = 0.064). The validated model for Hypotheses 1–3 is presented in Figure 2.

To examine H4 regarding the mediating role of self-efficacy in the relationship between badminton instructors’ leadership styles and exercise adherence intention, a bootstrap analysis using 5000 bootstrap samples with bias-corrected 95% confidence intervals was performed. The mediation results revealed that self-efficacy significantly mediated the relationship between training and instruction behavior and exercise adherence intention (standardized indirect effect = 0.141, 95% BC CI [0.025, 0.494], *p* = 0.014). The direct relationship between training and instruction behavior and exercise adherence intention was not significant, whereas the indirect relationship through self-efficacy was significant. However, self-efficacy did not mediate the relationships between democratic behavior, positive feedback behavior, or social support behavior and exercise adherence intention. Therefore, H4 was partially supported. The total effects of training and instruction, democratic, positive feedback, and social support behaviors on exercise adherence intention were 0.292, −0.184, 0.540, and −0.027, respectively. The R^2^ values were 0.509 for self-efficacy and 0.441 for exercise adherence intention.

The results for Hypothesis 4 are presented in Table 7.

## 5. Discussion

This study focused on recreational badminton participants who were taking badminton lessons. It examined the relationships between perceived badminton instructors’ leadership styles, self-efficacy, and exercise adherence intention, and investigated the mediating role of self-efficacy in the relationship between leadership styles and exercise adherence intention. The findings are discussed as follows.

First, H1, which proposed that badminton instructors’ leadership styles, specifically training and instruction, democratic, positive feedback, and social support behaviors, would have positive relationships with self-efficacy, was partially supported. Among the leadership-style dimensions, only training and instruction behavior was found to be significantly and positively related to self-efficacy. These findings suggest that clear technical instruction and feedback through training and instruction behavior may be associated with higher self-efficacy. The positive relationship between training and instruction behavior and self-efficacy is consistent with Bandura’s (1977) theory, which suggests that coaching behaviors can provide mastery experiences and may enhance participants’ self-efficacy. Similarly, Sari and Bayazit (2017) found that training and instruction behavior positively affected self-efficacy. This finding is consistent with the present study and suggests that coaching behaviors may strengthen participants’ psychological states and enhance their self-efficacy. In sports where technical skills and repetitive training are essential, an instructor’s training and instruction behavior may directly support performance development and perceived achievement. Therefore, specific training instructions, detailed feedback, and opportunities for successful experiences should be incorporated into coaching strategies to support participants’ self-efficacy.

Janas (2017), in a study of female adolescent basketball players, also reported a relationship between training and instruction behavior and self-efficacy, partially supporting the current findings. This suggests that when coaches clearly explain training methods and actively encourage participation, athletes may develop greater confidence in their ability to perform successfully. Furthermore, Unruh et al. (2019) reported that both training and instruction behavior and social support behavior were positively associated with student-athletes’ self-efficacy. These findings are partially consistent with those of our study, in which only training and instruction behavior was significantly and positively related to self-efficacy. Democratic, positive feedback, and social support behaviors were not significantly related to self-efficacy. Although conceptually distinct, Saville et al. (2014) suggested that coaches’ interaction behaviors may be critical factors in the development of participants’ self-efficacy. Similarly, Saville and Bray (2016) reported that coaching behaviors are positively associated with athletes’ self-efficacy.

Overall, training and instruction behavior may be related to higher levels of self-efficacy among badminton participants by providing technical learning experiences. The findings also suggest that participants may develop stronger self-efficacy through interactions with their instructors.

Second, H2, which proposed that badminton instructors’ leadership styles would have positive relationships with exercise adherence intention, was partially supported. Among the leadership-style dimensions, only positive feedback behavior was significantly and positively related to exercise adherence intention, whereas training and instruction, democratic, and social support behaviors were not significantly related to exercise adherence intention.

These results may reflect the characteristics of recreational badminton participation, where the intention to continue exercising may be more important than performance improvement. In this context, positive feedback and encouragement from instructors may help participants maintain positive exercise experiences and may be more closely related to their intention to continue exercising. Saville et al. (2014) and Saville and Bray (2016) suggested that coaches’ interactions and coaching behaviors are related to participants’ psychological responses. Annesi et al. (2011) also emphasized the importance of psychological factors in the relationship between coach-based training interventions and exercise adherence. These findings indicate the importance of instructor–participant interactions in relation to exercise adherence intention and support the present findings.

In contrast, training and instruction behavior, democratic behavior, and social support behavior were not significantly related to exercise adherence intention. This may suggest that, for recreational badminton participants, positive feedback and encouragement experienced during exercise may be more closely related to exercise adherence intention than technical instruction, participation in decision-making, or social support. Exercise adherence intention among recreational badminton participants may also be associated with other factors, such as social relationships, accessibility, cost, and exercise environment.

Third, H3, which proposed that self-efficacy would be positively related to exercise adherence intention, was supported. This finding aligns with McAuley and Blissmer (2000), who identified self-efficacy as a key factor explaining the initiation, maintenance, and long-term continuation of physical activity. The study by Hagger et al. (2001) involving adolescents also reported that self-efficacy is a critical predictor of physical activity intentions and behaviors. Previous studies consistently suggest that individuals with high self-efficacy tend to perceive exercise more positively and are more motivated to overcome participation barriers. Consequently, individuals with stronger self-efficacy are significantly more likely to maintain their intention to continue exercising. These findings align with the current results and indicate that self-efficacy is a key factor associated with stronger intentions to continue exercising.

Furthermore, Bélanger-Gravel and Godin (2010) identified self-efficacy as a significant determinant of children’s intentions to engage in physical activity and suggested that children who believe they can perform physical activity successfully, even in challenging situations, show stronger intentions to participate. This finding highlights the importance of helping children perceive physical activity as achievable and manageable. In clinical settings, Sniehotta et al. (2005) found that maintenance self-efficacy helped patients continue exercising during rehabilitation despite setbacks and temptations. Their findings indicate that confidence in one’s ability to maintain exercise behavior is a critical factor influencing exercise adherence.

Similarly, D’Angelo et al. (2007) reported a strong relationship between self-efficacy and exercise intention. They argued that simply recognizing the importance of exercise is insufficient; individuals must also believe they can overcome exercise-related barriers for their exercise intentions to increase. This is further supported by Depenbusch et al. (2022), who investigated physical activity, self-efficacy, exercise intention, and structural barriers among patients with cancer before and after diagnosis. Their findings indicated that structural barriers were associated with lower self-efficacy, which, in turn, was associated with lower exercise intention and physical activity levels. These results suggest a positive relationship between self-efficacy and exercise intention, implying that higher self-efficacy may be associated with stronger exercise intentions among patients. Woo et al. (2024) also reported that physical self-efficacy is associated with exercise persistence and commitment among certain groups of older adults, partially supporting this study’s findings.

Taken together, previous studies indicate that self-efficacy plays a critical role in sustaining physical activity and exercise participation. These findings support the current results. Therefore, strategies related to participants’ self-efficacy may be useful for maintaining their interest and engagement in exercise and supporting their intention to continue participating.

Fourth, H4, which proposed that self-efficacy would mediate the relationship between badminton instructors’ leadership styles and exercise adherence intention, was partially supported. Among the leadership-style dimensions, self-efficacy significantly mediated the relationship between training and instruction behavior and exercise adherence intention. This indirect effect should be interpreted cautiously because of its wide confidence interval. However, self-efficacy did not significantly mediate the relationships between democratic behavior, positive feedback behavior, or social support behavior and exercise adherence intention.

McAuley (1993) reported that self-efficacy is not only a critical predictor of exercise adherence among adults but may also serve as a significant mediating factor, thereby partially supporting this study’s findings. This suggests that higher self-efficacy may help maintain interest in exercise activities and promote long-term participation. Annesi et al. (2011) found that coach-based training interventions were related to exercise adherence outcomes through psychological variables, including self-efficacy. Similarly, Caetano et al. (2020) reported that self-efficacy and walking ability were key factors explaining continued exercise participation among older patients with stroke. Their findings suggest that combining coaching strategies that strengthen self-efficacy with interventions that improve actual physical abilities may be associated with enhanced exercise adherence.

J. Lee and Kim (2024) found that self-efficacy mediated the relationship between internet addiction and physical activity intentions among adolescents. Although their study involved a different population and variables, it supports the view that self-efficacy can function as an important factor linking antecedent factors to behavioral intentions. Furthermore, S. Y. Lee et al. (2024) identified a mediating effect of leisure satisfaction on the relationship between participation motivation and physical self-efficacy among sport climbing participants. They suggested that effective instructional methods, class structures, and feedback systems are necessary to promote sustained participation. Although their study used a different mediator, their findings emphasize the importance of the instructional environment, which is conceptually related to the present study.

Finally, Gong (2022) found that self-efficacy mediated the relationship between trust in coaches and exercise adherence intention among wrestlers. This finding aligns with the mediating role of self-efficacy identified in this study. Positive perceptions of instructors may be associated with greater confidence in participants’ abilities, which may, in turn, strengthen their intention to continue participating in exercise. These findings indicate that self-efficacy is a psychological factor associated with exercise adherence intention among recreational badminton participants. Theoretically, SCT explains how instructor leadership may relate to participants’ self-efficacy, while TPB explains how self-efficacy may relate to exercise adherence intention (Ajzen, 1991; Bandura, 1977). Together, these theories help explain the mediating role of self-efficacy observed in this study.

## 6. Conclusions

This study focused on recreational badminton lesson participants and provides foundational evidence on exercise adherence intention in this population. The study examined the relationships between perceived badminton instructors’ leadership styles, self-efficacy, and exercise adherence intention. Specifically, the structural relationships among leadership styles, self-efficacy, and exercise adherence intention were investigated, and the mediating role of self-efficacy in the relationship between leadership styles and exercise adherence intention was examined.

The main findings are summarized as follows.

First, among the sub-dimensions of badminton instructors’ leadership styles, training and instruction behavior had a significant positive relationship with self-efficacy, whereas democratic behavior, positive feedback behavior, and social support behavior were not significantly related to self-efficacy.

Second, positive feedback behavior had a significant positive relationship with exercise adherence intention, whereas training and instruction behavior, democratic behavior, and social support behavior were not significantly related to exercise adherence intention.

Third, self-efficacy had a significant positive relationship with exercise adherence intention.

Fourth, self-efficacy significantly mediated the relationship between training and instruction behavior and exercise adherence intention. However, self-efficacy did not significantly mediate the relationships between democratic behavior, positive feedback behavior, or social support behavior and exercise adherence intention.

## 7. Implications and Limitations

Based on these findings, the following implications and limitations are presented.

First, this study provides practical insights for badminton coaching by identifying leadership styles related to participants’ self-efficacy and exercise adherence intention. These findings suggest that self-efficacy-focused coaching strategies may complement technical instruction. Such strategies include considering individual characteristics, establishing clear performance and learning goals, and providing feedback appropriate to participants’ skill levels. Future studies should develop educational or field-based intervention programs to further examine the effectiveness of different leadership styles.

Second, because badminton is widely practiced across diverse age groups, cultural backgrounds, and social environments, perceptions of leadership styles may vary across cultural and regional contexts. Distinct social norms, sport participation cultures, and coaching practices may lead participants to perceive and respond to the same leadership style differently. While this study examined the relationships among variables using a quantitative approach, future research should employ qualitative or mixed-methods research designs to gain a deeper understanding of how participants perceive and experience different leadership styles. Such efforts may contribute to a more comprehensive understanding of cultural differences and enhance the interpretation and generalizability of the findings.

Third, because this study employed a cross-sectional design, causal relationships among leadership styles, self-efficacy, and exercise adherence intention could not be established. The use of a self-report survey may also have introduced common-method bias and reverse causality. Convenience sampling and the restriction of the sample to lesson participants may also limit the generalizability of the findings. In addition, 49.5% of the participants were in their 30s, which may limit the generalizability of the findings to other age groups. Some participants may have shared the same instructor or facility, potentially introducing clustering. Because instructor- and facility-level identifiers were not collected, this potential clustering could not be examined. This study measured general self-efficacy, reflecting broader beliefs about one’s ability to manage difficulties and perform necessary behaviors across situations. Future studies could examine exercise-specific self-efficacy and compare its role with that of general self-efficacy in exercise or badminton contexts. In addition, autocratic behavior was excluded from the final model based on the measurement validation results, representing a measurement-related limitation. Future studies should further examine this leadership dimension in different samples. Shared variance among leadership dimensions may also have influenced the individual path coefficients. This study focused on exercise adherence intention. Future studies should consider other exercise behaviors, such as actual participation frequency and duration. Future studies should employ longitudinal or experimental designs to examine how changes in instructors’ leadership styles are related to participants’ self-efficacy and long-term exercise adherence over time. Such approaches would provide stronger evidence regarding the causal mechanisms underlying sustained exercise participation.

## Figures and Tables

**Table 1:** Participants’ demographic characteristics.

Variable	Category	Frequency (N)	Percentage (%)
Gender	Male	118	63.4
	Female	68	36.6
Age	Under 20 years	5	2.7
	20–29 years	26	14.0
	30–39 years	92	49.5
	40–49 years	28	15.1
	50–59 years	27	14.5
	60 years and above	8	4.3
Occupation	Students	25	13.4
	Homemaker	5	2.7
	Office worker	116	62.4
	Self-employed	11	5.9
	Public official	6	3.2
	Professional	13	7.0
	Other	10	5.4
Badminton Participation Frequency	One to two times per week	64	34.4
	Three to four times per week	61	32.8
	Five to six times per week	49	26.3
	Seven times per week	12	6.5
Duration per Session	Less than 1 h	20	10.8
	1–2 h	83	44.6
	3–4 h	63	33.9
	5 h or more	20	10.8

**Table 2:** Factor and Reliability Analysis of Badminton Instructors’ Leadership Styles.

Factor	Item	Standardized Loading	S.E.	C.R.	*p*	Cronbach’s α
Training and Instruction Behavior	(My badminton instructor) demonstrates techniques in detail.	0.788				0.859
	uses various materials and methods for instruction.	0.773	0.099	10.885	***	
	accurately identifies my strengths and weaknesses.	0.783	0.095	11.046	***	
	corrects my mistakes immediately.	0.781	0.078	11.010	***	
Democratic Behavior	(My badminton instructor) allows me to suggest practice methods.	0.777				0.844
	allows me to set my own goals.	0.842	0.093	11.749	***	
	allows me to participate in decision-making.	0.790	0.095	10.992	***	
Positive Feedback Behavior	(My badminton instructor) rewards me according to my performance.	0.893				0.879
	gives me a lot of praise.	0.891	0.062	17.236	***	
	treats me favorably.	0.758	0.057	12.846	***	
Social Support Behavior	(My badminton instructor) tries to gain my trust.	0.734				0.847
	talks with me frequently.	0.892	0.095	11.717	***	
	shows concern for my personal welfare.	0.816	0.085	10.841	***	

Note: Model fit: χ^2^ = 123.039, df = 59, *p* < 0.001, CMIN/df = 2.085, GFI = 0.904, AGFI = 0.851, CFI = 0.960, NFI = 0.927, RMR = 0.034, RMSEA = 0.077. *** *p* < 0.001.

**Table 3:** Factor and Reliability Analysis of Self-Efficacy.

Factor	Item	Standardized Loading	S.E.	C.R.	*p*	Cronbach’s α
Self-Efficacy	I can persist and work hard without giving up easily.	0.744				0.903
	I can make full use of available information to accomplish a given task.	0.874	0.099	11.983	***	
	I can analyze the causes and consequences of a situation effectively.	0.828	0.102	11.333	***	
	I have the ability to overcome difficult situations.	0.746	0.097	10.125	***	
	I am confident in myself.	0.732	0.105	9.914	***	
	My judgment about problems is generally accurate.	0.752	0.100	10.207	***	

Note: Model fit: χ^2^ = 17.176, df = 9, *p* = 0.046, CMIN/df = 1.908, GFI = 0.971, AGFI = 0.933, CFI = 0.987, NFI = 0.973, RMR = 0.017, RMSEA = 0.070. *** *p* < 0.001.

**Table 4:** Factor and Reliability Analysis of Exercise Adherence Intention.

Factor	Item	Standardized Loading	S.E.	C.R.	*p*	Cronbach’s α
Exercise Adherence Intention	I will continue playing badminton even when I am under emotional stress.	0.917				0.921
	I will continue playing badminton even when I feel physically tired.	0.914	0.052	18.464	***	
	I will continue playing badminton even when I have limited time.	0.846	0.052	16.202	***	

Note: Model fit: χ^2^ = 0.000, df = 0 (saturated model). *** *p* < 0.001.

**Table 5:** Correlations, Construct Reliability, and Average Variance Extracted (AVE).

Factors	1	2	3	4	5	6
1	1					
2	0.643	1				
3	0.679	0.720	1			
4	0.589	0.653	0.764	1		
5	0.585	0.522	0.580	0.578	1	
6	0.491	0.421	0.545	0.449	0.555	1
Construct Reliability	0.862	0.845	0.886	0.857	0.904	0.922
AVE	0.610	0.646	0.722	0.667	0.612	0.798
√AVE	0.781	0.804	0.850	0.817	0.782	0.893

Note: 1. Training and instruction behavior; 2. Democratic behavior; 3. Positive feedback behavior; 4. Social support behavior; 5. Self-efficacy; 6. Exercise adherence intention; √AVE = square root of the average variance extracted.

**Table 6:** Results of Hypotheses 1–3.

Hypothesis	Path	Standardized Coefficient	S.E	C.R	*p*	Result
1-1	Training and Instruction Behavior → Self-Efficacy	0.396	0.121	3.190	0.001 **	Accepted
1-2	Democratic Behavior → Self-Efficacy	0.032	0.110	0.217	0.828	Rejected
1-3	Positive Feedback Behavior → Self-Efficacy	0.055	0.194	0.275	0.783	Rejected
1-4	Social Support Behavior → Self-Efficacy	0.297	0.132	1.890	0.059	Rejected
2-1	Training and Instruction Behavior → Exercise Adherence Intention	0.150	0.171	1.113	0.266	Rejected
2-2	Democratic Behavior → Exercise Adherence Intention	−0.196	0.151	−1.229	0.219	Rejected
2-3	Positive Feedback Behavior → Exercise Adherence Intention	0.521	0.274	2.403	0.016 *	Accepted
2-4	Social Support Behavior → Exercise Adherence Intention	−0.134	0.185	−0.789	0.430	Rejected
3	Self-Efficacy → Exercise Adherence Intention	0.358	0.133	3.487	***	Accepted

* *p* < 0.05, ** *p* < 0.01, *** *p* < 0.001.

**Table 7:** Results of Hypothesis 4.

Hypothesis	Path	Standardized Coefficient	Indirect Effect		*p*	Result
			Lower	Upper		
4-1	Training and Instruction Behavior → Self-Efficacy → Exercise Adherence Intention	0.141	0.025	0.494	0.014 *	Accepted
4-2	Democratic Behavior → Self-Efficacy → Exercise Adherence Intention	0.012	−0.110	0.200	0.731	Rejected
4-3	Positive Feedback Behavior → Self-Efficacy → Exercise Adherence Intention	0.020	−0.209	0.232	0.769	Rejected
4-4	Social Support Behavior → Self-Efficacy → Exercise Adherence Intention	0.106	−0.048	0.432	0.178	Rejected

* *p* < 0.05.

## Data Availability

The original contributions presented in this study are included in the article. Further inquiries can be directed to the corresponding author.
